# Mechanical power distribution of the lower limbs changed during intermittent 300 countermovement jumps

**DOI:** 10.1007/s00421-024-05619-8

**Published:** 2024-09-26

**Authors:** Maximilian Sanno, Jan-Peter Goldmann, Kai Heinrich, Patrick Wahl, Gert-Peter Brüggemann

**Affiliations:** 1 Institute of Biomechanics and Orthopaedics, German Sport University Cologne, Am Sportpark Müngersdorf 6, 50933 Cologne, Germany; 2 German Research Center of Elite Sport, German Sport University Cologne, Am Sportpark Müngersdorf 6, 50933 Cologne, Germany; 3 Institute of Cardiology and Sports Medicine, German Sport University Cologne, Am Sportpark Müngersdorf 6, 50933 Cologne, Germany

**Keywords:** Kinetics, Contribution, Fatigue, Knee, Ankle

## Abstract

**Purpose:**

The aim of this study was to investigate the effect of 300 intermittent countermovement jumps (CMJs) on the mechanical power distribution at the joints of the lower limbs and the influence of the upper body to explain vertical jump performance.

**Methods:**

Fifteen male sport students (age 24.5 ± 2.3 years; body height 1.85 ± 0.06 m; body mass 84.8 ± 8.5 kg) performed a set of intermittent 300 CMJs at maximal effort. An inverse-dynamic approach was used to calculate the mechanical power at the hip, knee, and ankle joint for each jump.

**Results:**

Jump height and mechanical power in the knee and ankle joints decreased significantly (*p* < .010), while remained the same in the hip joint. In contrast, a significant increased vertical velocity was observed for the upper body segment. In addition, a significant higher angular momentum at the center of mass was detected during the braking and propulsion phase.

**Conclusion:**

The findings highlight a fatigue-related decrease in lower limb power, particularly in the knee and ankle joints, which changed the mechanical power distribution at the joints of the lower limbs. The trunk extensor muscles were probably able to counteract the fatigue-related decrease in lower limb power by increased vertical velocity of the upper body segment and higher angular momentum at the center of mass during the braking and propulsion phase. Accordingly, the most effective way to maintain jumping performance in fatigued state would be to improve the fatigue resistance of the knee extensors, ankle plantar flexors, and trunk extensor muscles.

## Introduction

The mechanical output of lower limbs is important for many sports requiring explosive muscle action, and is often determined by vertical jump performance of countermovement jumps (CMJs) (Smirniotou et al. 2008; Bobbert and van Soest 1994, 2001; Jacobs et al. 1996). In general, vertical jump performance is measured by the maximum vertical displacement of the center of mass (CoM), which is a result of the vertical impulse at take-off and is associated with the vertical mechanical peak power at the CoM (Dowling and Vamos 1993; Harman et al. 1990; Aragón-Vargas and Gross 1997). The vertical mechanical power at the CoM arises from multi-segment joint movements of the total body, with different mechanical contributions from both the upper body and the lower limbs, resulting of the transfer of torques of the involved joints to the ground (Yamauchi and Ishii 2007; Vanezis and Lees 2005; Bobbert 2001; Butcher et al. 2007; Mills et al. 2005). It has been shown that the rotation of the upper body segment can contribute about 10% to vertical jump height (Vanrenterghem et al. 2008; Luhtanen and Komi 1978).

Many studies have examined the effects of joint-specific contribution of ankle, knee, and hip extensors to vertical jump height, which can be indicated by joint torque, power, and work (Hubley and Wells 1983; Fukashiro and Komi 1987; Robertson and Fleming 1987; Pandy and Zajac 1991; Aragón-Vargas and Gross 1997; Vanezis and Lees 2005; Bobbert et al. 1986; Bobbert and van Ingen Schenau 1988; Bobbert and van Soest 1994; Lees et al. 2004). In particular, the knee and ankle joints’ muscle capacities are more important for jump height than those of the hip extensors (Nagano and Gerritsen 2001; Cheng 2008). However, there are controversial results in the relative contribution of lower limb extensors to jump height during CMJs, which is reflected in an inconsistent contribution of hip or knee extensors (Robertson and Fleming 1987; Hubley and Wells 1983; Fukashiro and Komi 1987). From a mechanistic approach, not only the relative joint contributions are of interest for the description of vertical jumping, but also the effective mechanical advantage (EMA) of the joints of the lower limbs (Farris et al. 2016). The EMA is defined as the ratio (*r/R*) of internal lever arm (*r*) which is the perpendicular distance between the center of the joint and the tendon line of action, and external lever arm (*R*) which describes the leverage characteristics of an investigated joint (Biewener et al. 2004). Changes in the ability of muscle to produce power could be explained, among others, by differences in EMA and the required active muscle volume for a defined task (Roberts and Marsh 2003; Biewener et al. 2004; Farris et al. 2016).

Research on maximum vertical jump performance has so far mainly focused on the non-fatigued state, but in many sports, fatigue is an important factor in athletic performance. In general, fatigue is an exercise-induced reduction in the ability of the muscle to produce force or power, whether or not the task can be sustained (Bigland-Ritchie and Woods 1984; Søgaard et al. 2006; Bigland-Ritchie et al. 1983; Barry and Enoka 2007; Gandevia 2001). There are currently two main terminologies that are commonly used to classify fatigue: (i) peripheral (muscular) fatigue, which is defined as an impairment of the force-generating capacity of the muscle, and (ii) central (neural) fatigue, which is defined as the failure in neuronal drive resulting decreased ability to send a signal to the neuromuscular junction (Maclaren et al. 1989; Gandevia 2001; Enoka and Duchateau 2008). Studies on central fatigue generally focus on isometric contractions of isolated muscle groups using the twitch interpolation technique (Bigland-Ritchie et al. 1986; Löscher et al. 1996; Hortobágyi et al. 1996). More recent studies have also investigated fatigue induced by more complex functional movements such as running or jumping (Saldanha et al. 2008; Sanno et al. 2018; Rodacki et al. 2001; Millet et al. 2003). When jumping, it seems certain that muscular fatigue decreases jump height, as well as peak joint angular velocity, peak joint net torque, and peak joint power of the lower limbs, which is often demonstrated in fatiguing of individual muscle groups, or continuous or intermittent jumping (Rodacki et al. 2001, 2002; McNeal et al. 2010; Bosco et al. 1983; Fábrica et al. 2013; Bobbert et al. 2011; Pereira et al. 2014).

To the best of our knowledge a single fatiguing protocol (maximum number of intermittent CMJs with a minimum target height of 95% of the maximum jump height) has been implemented to investigate the effects of fatigue on jumping mechanics (Pereira et al. 2014, 2009a, 2009b). In this context, Pereira et al. (2008) demonstrated that a short rest (8 s) leads to a significant reduction in the number of CMJs compared to longer rests (≥ 14 s), possibly due to an increased physical load on the cardiovascular system and an increased lactate concentration or acidosis which may inhibit muscle contractile activity or indicate a state of fatigue (Cairns 2006; Fitts 1994; Gaesser and Poole 1996). However, no significant changes of the segmental contribution and intersegmental coordination were found during such intermittent vertical jumps performed until fatigue based of sagittal plane lower limb joint angles during the eccentric and concentric phases of the CMJ (Pereira et al. 2014). Moreover, Pereira et al. (2009b) found that intermittent CMJs using the same resting periods lead to a similar (7%) decrease in maximal voluntary isometric knee extension torque.

Nevertheless, it is currently unknown whether a jump-induced fatigue changes the kinetic contributions of lower limb extensors or which muscle group of the lower limbs is more likely to be affected by a jump-induced reduction in the ability to generate mechanical joint power, and whether this reduction influences the mechanical contributions of the upper body segment.

Therefore, the purpose of this study was to investigate the effect of 300 intermittent countermovement jumps on mechanical power distribution at the joints of the lower limbs and the influence of the upper body to explain vertical jump performance. It was hypothesized that an intermittent jump-induced fatigue protocol decreases the CMJ height, which would be primarily explained by a larger decrease in mechanical joint power of the knee extensors and ankle plantar flexors than the hip extensors or the vertical velocity of the upper body segment.

## Methods

### Subjects

Fifteen well-trained male sport students (age 24.5 ± 2.3 years; body height 1.85 ± 0.06 m; body mass 84.8 ± 8.5 kg) were recruited. All participants were injury-free for the 12 months prior to data collection and signed a written informed consent form before participation.

### Design

In a cross-sectional study design, all participants performed a set of 300 CMJs at maximal effort (without arm swing, hands on hips) within 40 min to achieve jump-induced fatigue. The participants were given a fixed rest interval (8 s) between each jump (Pereira et al. 2008, 2009b) which was controlled by a clock generator for acoustic signals. Before the set of 300 CMJs, the participants executed warm-up exercises with self-determined duration. During the 300 CMJs, the participants were continuously encouraged and informed about the number of CMJs completed.

**Fig. 1:**
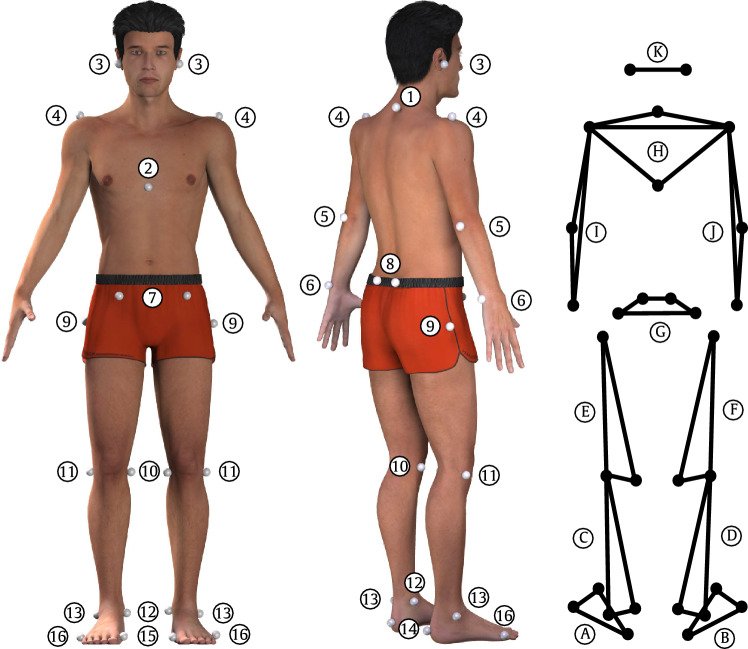
Schematic illustration of the marker position (left and middle). A total of 30 spherical reflective markers (Ø13 mm, Ilumark GmbH; Feldkirchen/Munich, Germany) were attached to the following bony landmarks: (1) 7th cervical vertebra, (2) distal point of sternum, and to attend to left and right: (3) os temporale of the head, (4) top of acromion, (5) lateral humeral epicondyle, (6) distal lateral ulnar head, (7) spina iliaca anterior and (8) posterior, (9) greater trochanter, (10) medial and (11) lateral femoral condyles, (12) medial and (13) lateral malleoli, (14) posterior aspect of the heel, and (15) first and (16) fifth metatarsal heads. The markers of the pelvis (7–9) were attached on the skin. The markers of the foot (12–16) were attached over the anatomical landmarks on the upper of the shoe. The three-dimensional inverse dynamical model of the total body (right) consisting of 11 segments divided in lower body: (A) left and (B) right of foot, (C, D) shank, (E, F) thigh, and (G) pelvis, and upper body: (H) trunk, (I, J) both arms, and (K) head

Each of the 300 CMJs was captured with a 11 infrared camera motion capture system (120 Hz, MX-F40, Vicon Motion Systems^™^; Oxford, UK) synchronized with two force plates (model 9287, 1080 Hz, Kistler Instrumente AG; Winterthur, Switzerland). Before the set of 300 CMJs, 30 markers were attached to the bony landmarks (Fig. 1). All marker coordinates and the ground reaction force data were smoothed by using a recursive fourth-order Butterworth low-pass filter (cut-off frequency: 15 Hz) (Bobbert et al. 1987a, 1987b).

A three-dimensional inverse dynamics model of the total body (alaska^®^ Dynamcius^®^ 7.0, Institute of Mechatronics; Chemnitz, Germany) consisting of 11 segments (Fig. 1) was used to calculate the kinematic and kinetic data. Individual body height and mass were imported to the model to obtain anthropometric dimensions and inertial properties for each segment. The net joint torque was referenced to the anatomical coordinate system of the proximal segment of the joint. A reference trial was recorded in an upright position to determine the neutral position of all joints (0° joint angle). In addition, all kinetic parameters for the CoM were determined using the sum vector of both ground reaction forces. Customized MATLAB routines (MathWorks Inc., Natick, MA) were used to determine all kinematic and kinetic parameters.

### Parameters

For parameter calculation, a countermovement jump was divided into following phases: weighting, unweighting, breaking, and propulsion phase (McMahon et al. 2018; Linthorne 2001). The time of breaking phase begins at the end of the unweighting phase and ends when COM velocity equals zero (CoM_v0_). The time of propulsion phase begins when CoM velocity becomes positive and ends at take-off.

The maximal vertical ground reaction force (GRF_Max_) during the CMJ and the maximal rate of vertical force development at the CoM (RFD_CoM_) during the propulsion phase were determined. Maximum (maxP_CoM_) and mean (P_CoM_) mechanical power at the CoM during the propulsion phase were calculated by the integration of vertical ground reaction force. The jump height is subsequently determined using the impulse–momentum method (Linthorne 2001).

The individual CoM vertical velocity of lower body and total body was determined as well as the difference between these two velocities, resulting in the individual vertical CoM velocity of the upper body segment. Thereafter, the maximum of angular momentum at the CoM of total body was determined between the beginning of the CMJ and the end of the breaking phase (AM_Breaking_) as well as for the propulsion phase (AM_Propulsion_). During the propulsion phase, also the mean angular velocities of the hip, knee, and ankle joint were calculated. For the CoM_v0_, the hip, knee, and ankle joint angles, external lever arms (corresponds to the distance between the center of the joint and the ground reaction force vector), and external torques (product of the external lever arm and the ground reaction force vector) were determined. The EMA was determined for the joints of the lower limbs during the CoM_v0_. To calculate the EMA, the length of the hip, knee, and ankle joint internal lever arms were taken from the literature and used uniformly for all participants (Biewener et al. 2004). The internal muscle force (*F*_*m*_) was estimated using the quotient of GRF and EMA during the CoM_v0_. Active agonist muscles responsible for jumping were considered to be the hamstrings and gluteus maximus at the hip, the quadriceps at the knee, and the triceps surae at the ankle. The volume of these active muscles (*V*_*m*_) was calculated separately for each joint (Eq. 1). The assumption is that the muscles produced force isometrically with a constant stress (*σ* = 20 N·cm^−2^) (Perry et al. 1988) and combined this with our estimates of *F*_*m*_ and weighted-average fascicle lengths (*L*) from Biewener et al. (2004):

$$V_{m} = \frac{{F_{m} \cdot L}}{\sigma }$$

During the propulsion phase, mean mechanical power at hip (P_Hip_), knee (P_Knee_), and ankle joint (P_Ankle_) was calculated as the product of the individual joint torque and joint angular velocity. In addition, the sum of all joints (P_Sumjoints_) was determined. The positive work at the hip, knee, and ankle joint was obtained by numerical integration of the corresponding power-time curve.

All analyses were performed within the sagittal plane and all parameters are presented as mean and standard deviation (SD). Joint angles, angular velocities, and external lever arms are presented as mean of left and right, whereas external joint torques, power, and work demonstrated as sum of left and right as well as normalized to body mass.

In addition to biomechanical measures, also heart rate was recorded throughout the whole jumping protocol by a heart rate monitor (M51, Polar Electro; Kempele, Finland). Furthermore, capillary samples from the earlobe were collected for blood lactate concentration analysis (EBIOplus, EKF Diagnostic Sales; Magdeburg, Germany) prior the beginning, after 100 CMJs, 200 CMJs, and 300 CMJs during the 8 s rest interval.

### Statistical analysis

A univariate analysis of variance (ANOVA) with repeated measures was used to detect potential changes in heart rate and blood lactate concentration in four time points: prior the beginning, after 100 CMJs, 200 CMJs, and 300 CMJs. To determine possible changes in the jump height, a further repeated-measures ANOVA was used to analyze the averages of 20 CMJs in 15 continuous sets. In case of a significant effect a Bonferroni *post-hoc* correction was applied to detect the significant differences. In addition, for all parameters, paired one-tailed Student's t tests were performed between the initial 20 CMJs (CMJ_Start_) and the final 20 CMJs (CMJ_End_). Cohen’s *d* effect sizes (1988. p. 40, 286–7) were calculated to explain the strength of a phenomenon (small effect size *d* ≤ 0.200; medium *d* ≤ 0.500; large *d* ≤ 0.800). The level of significance was defined as α = 0.050. All statistical analyses were performed using SPSS Statistics 23 (IBM Corporation; New York, USA).

## Results

Between the beginning and the end of the 300 CMJs, heart rate increased significantly (*p* < 0.001, *d* = 12.778) from 82.0 ± 8.1 beats per minute (BPM) to 172.5 ± 11.2 BPM. The blood lactate concentration increased significantly (*p* < 0.001, *d* = 1.407) from 1.14 ± 0.32 mmol·l^−1^ to 3.44 ± 1.53 mmol·l^−1^.

**Fig. 2:**
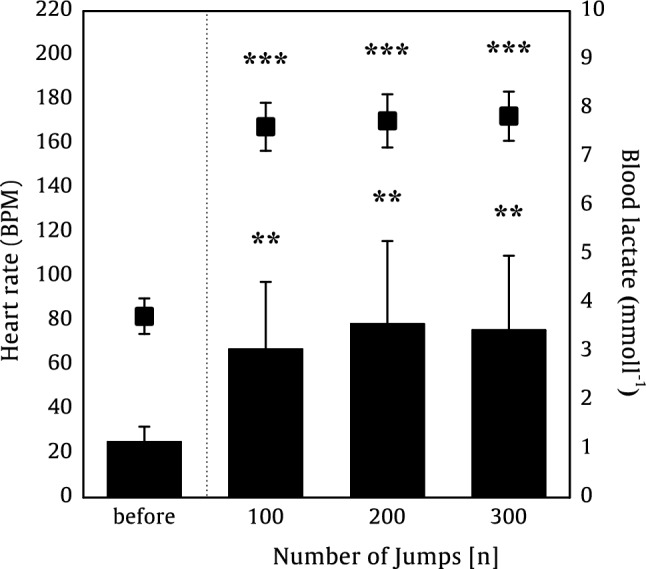
Heart rate (filled squares) and blood lactate concentration (bars) as a mean ± SD of 15 participants before, after 100, 200, and 300 CMJs. Significant differences between before the set of 300 CMJ and the 100 CMJ, 200 CMJ, and 300 CMJ are represented by **p < .010 and ***p < .001

**Fig. 3:**
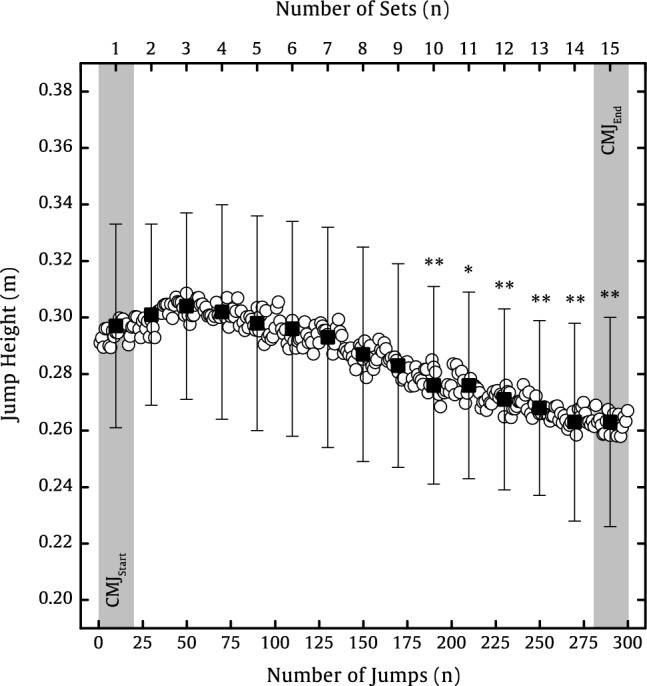
Jump height as a mean of 15 participants for all 300 CMJs (circles), and 15 sets as a mean ± SD of 20 CMJs (filled squares). CMJ_Start_ represents the initial 20 CMJs (1st set), and CMJ_End_ represents the final 20 CMJs (15^th^ set). Significant differences to the CMJ_Start_ are represented by *p < .050 and **p < .010

Jump height decreased significantly (*p* < 0.001, *d* = 1.745) about 11% between CMJ_Start_ (0.297 ± 0.036 m) and CMJ_End_ (0.263 ± 0.037 m) as well as about 13% between CMJ at highest set (No.3; *p* < 0.001, *d* = 2.334; 0.304 ± 0.033 m) and the end (Fig. 2). Based on the inverse dynamics model, the maximal CoM velocity of lower limb segment decreased significantly (*p* < 0.001, *d* = 1.131) about 4% between CMJ_Start_ (2.45 ± 0.16 m·s^−1^) and CMJ_End_ (2.34 ± 0.17 m·s^−1^). In contrast, the maximal vertical velocity of upper body segment increased significantly (*p* < 0.001, *d* = − 0.699) about 9% between CMJ_Start_ (0.84 ± 0.07 m·s^−1^) and CMJ_End_ (0.91 ± 0.14 m·s^−1^) (Fig. 3).

Joint angles, angular velocities, lever arms, EMA, torques, power, and work of the CMJ_Start_ and CMJ_End_ are represented in Table 1. The estimated internal muscle force of the lower limbs decreased significantly for the hip (*p* = 0.036, *d* = 0.504) about 8%, knee (*p* < 0.001, *d* = 1.208) about 18%, and ankle (*p* = 0.003, *d* = 0.847) about 16%. The estimated relative proportion of active muscle volume at the hip increased from 60 to 62% of the total volume, while it decreased at the knee (25–24%) and ankle (15–14%).

**Table 1:** Kinematic and kinetic parameters as means ± SD of the initial (CMJ_Start_) and final 20 CMJs (CMJ_End_) of 300 CMJs

	CMJ_Start_			CMJ_End_			*p*	*d*	Change
Time									
(s) t_Breaking_	0.181	±	0.020	**0.220**	** ± **	0.029***	< .001	1.357	+ 22%
t_Propulsion_	0.301	±	0.033	**0.343**	** ± **	0.050***	< .001	1.093	+ 14%
Force									
(N·kg^−1^) GRF_Max_	13.60	±	1.61	**11.82**	** ± **	1.68***	< .001	1.051	− 13%
(N·s^−1^) RoFD_CoM_	6189	±	1497	**4188**	** ± **	1134***	< .001	1.376	− 32%
Power									
(W·kg^−1^) maxP_CoM_	44.35	±	3.97	**41.79**	±	5.08***	< .001	1.064	− 6%
P_CoM_	24.88	±	2.10	**21.47**	±	2.30***	< .001	1.903	− 14%
Angular momentum									
(kgm^2^·s^−1^) AM_Breaking_	0.129	±	0.020	**0.143**	** ± **	0.035*	0.038	0.493	+ 11%
AM_Propulsion_	0.138	±	0.032	**0.162**	** ± **	0.058**	0.006	0.748	+ 18%
Joint angle at CoM_v0_ (mean of left and right)									
(°) Hip	95.8	±	11.8	**100.5**	** ± **	10.9*	0.025	0.556	+ 5%
Knee	104.2	±	9.9	106.1	±	12.8	0.229	0.197	+ 2%
Ankle	29.8	±	5.0	28.8	±	5.7	0.090	0.365	− 3%
Mean angular velocity_Propulsion_ (mean of left and right)									
(°·s^−1^) Hip	315.4	±	28.5	**293.7**	** ± **	30.6***	< 0.001	1.170	− 7%
Knee	341.3	±	23.3	**305.1**	** ± **	33.4***	< 0.001	1.738	− 11%
Ankle	240.7	±	22.0	**211.2**	** ± **	25.0***	< 0.001	1.181	− 12%
Lever arm at CoM_v0_ (mean of left and right)									
(m) Hip	0.237	±	0.021	**0.251**	** ± **	0.032*	0.015	0.624	+ 6%
Knee	0.153	±	0.030	0.146	±	0.036	0.076	0.392	− 5%
Ankle	0.108	±	0.029	0.105	±	0.030	0.162	0.263	− 3%
EMA at CoM_v0_ (mean of left and right)									
(r/R) Hip	0.243	±	0.021	**0.231**	** ± **	0.031*	0.039	0.489	− 5%
Knee	0.371	±	0.071	0.401	±	0.116	0.087	0.369	+ 8%
Ankle	0.378	±	0.158	0.392	±	0.151	0.296	0.142	+ 3%
External joint torque at CoM_v0_ (sum of left and right)									
(Nm·kg^−1^) Hip	2.03	±	0.27	2.05	±	0.35	0.388	0.075	+ 1%
Knee	2.27	±	0.28	**2.02**	** ± **	0.33***	< 0.001	1.554	− 11%
Ankle	2.21	±	0.18	**2.04**	** ± **	0.21***	< 0.001	1.032	− 8%
Mean joint power_Propulsion_ (sum of left and right)									
(W·kg^−1^) Hip	7.19	±	1.36	6.97	±	1.66	0.207	0.217	− 3%
Knee	9.25	±	1.49	**7.84**	** ± **	1.59***	< 0.001	1.444	− 15%
Ankle	6.63	±	0.88	**5.52**	** ± **	0.99***	< 0.001	1.093	− 17%
Sum	23.07	±	2.29	**20.33**	** ± **	2.60***	< 0.001	1.537	− 12%
Joint work_Pos_ (sum of left and right)									
(W·kg^−1^) Hip	2.69	±	0.50	2.83	±	0.57	0.272	0.296	+ 5%
Knee	2.93	±	0.54	2.81	±	1.59	0.220	0.332	− 4%
Ankle	1.99	±	0.21	**1.89**	** ± **	0.25*	0.012	0.748	− 5%
Sum	7.62	±	0.89	7.52	±	0.76	0.508	0.175	− 1%

The time of breaking phase (t_Breaking_) begins at the end of the unweighting phase and ends when center of mass velocity equals zero (CoM_v0_). The time of propulsion phase (t_Propulsion_) begins when CoM velocity becomes positive and ends at take-off. The maximal vertical ground reaction force (GRF_Max_), rate of vertical force development at the CoM (RFD_CoM_), as well as maximum (maxP_CoM_) and mean (P_CoM_) mechanical power at the CoM during the propulsion phase. The maximum of angular momentum at the CoM between the beginning of the CMJ and the breaking phase (AM_Breaking_) and for the propulsion phase (AM_Propulsion_) as well as angle, angular velocity, external lever arm, EMA, net torque, power, and positive work of the hip, knee, and ankle joint. Significant differences are represented by *p < .050, *p < .010, and ***p < .001, as well as *p *values, Cohen’s *d* effect sizes, and the percentage changes between CMJ_Start_ and CMJ_End_ are shown

**Fig. 4:**
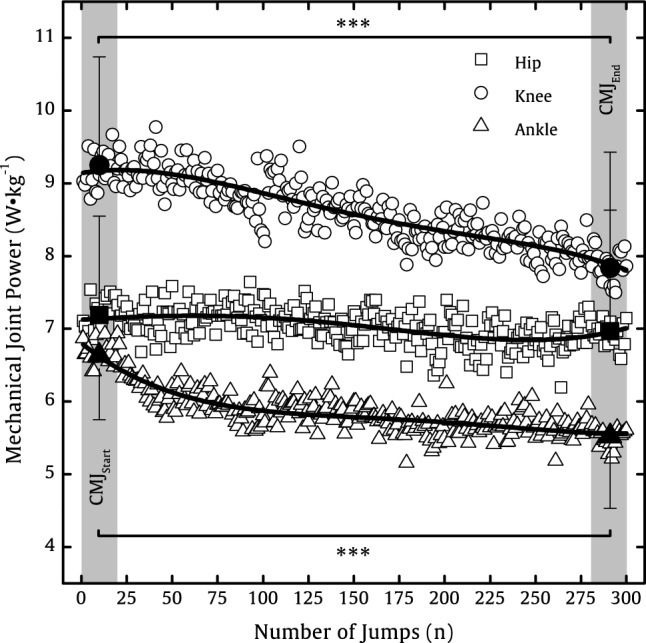
Mechanical power at the hip (squares), knee (circles), and ankle joint (triangles) as a mean of 15 participants for all 300 CMJs. The first set as a mean ± SD of initial 20 CMJs (CMJ_Start_) and the last set as a mean ± SD of final 20 CMJs (CMJ_End_) are represented by filled squares, triangles, and circles. Significant differences between CMJ_Start_ and CMJ_End_ are represented by ***p < .001

The P_Hip_, P_Knee_, and P_Ankle_ for all 300 CMJs are represented in Fig. 4. The joint-specific contribution of mechanical power to the P_Sumjoints_ was for CMJ_Start_ (hip: 31.2%; knee: 40.1%; ankle: 28.7%) and for CMJ_End_ (34.3%; 38.5%; 27.2%). The Pearson product–moment correlation coefficients between the ΔHeight, ΔP_CoM_, ΔP_Hip_, ΔP_Knee_, ΔP_Ankle_, and ΔP_Sumjoints_ are represented in Table 2. The ∆P_Sumjoints_ was in the mean of the group − 2.733 ± 1.778 W·kg^−1^ and made up of 8% ∆P_Hip_, 52% ∆P_Knee_, and 40% ∆P_Ankle_.

**Table 2:** Pearson product–moment correlation coefficient matrix of the difference (∆) between CMJ_Start_ and CMJ_End_

	Correlations					
	ΔHeight	ΔP_CoM_	ΔP_Hip_	ΔP_Knee_	ΔP_Ankle_	ΔP_Sumjoints_
ΔHeight	1					
ΔP_CoM_	0.742**	1				
ΔP_Hip_	0.465	0.342	1			
ΔP_Knee_	0.254	0.528*	− 0.401	1		
ΔP_Ankle_	0.299	0.781**	0.000	0.488	1	
ΔP_Sumjoints_	0.572*	0.927**	0.345	0.601*	0.837**	1

*Note.* Pearson product–moment correlation coefficient matrix of the jump height as the difference (∆) between CMJ_Start_ and CMJ_End_ (ΔHeight) and the mechanical power at the center of mass of the total body (ΔP_CoM_), as well as mechanical power at hip (ΔP_Hip_), knee (ΔP_Knee_), and ankle joint (ΔP_Ankle_), as well as the sum of mechanical power of all three joints of the lower body (ΔP_Sumjoints_). ΔP_Hip_, ΔP_Knee_, and ΔP_Ankle_ are the sum of left and right. Significant differences are represented by *p < .050 and *p < .010 (two-tailed)

## Discussion

The purpose of this study was to investigate the effect of 300 intermittent countermovement jumps on mechanical power distribution at the joints of the lower limbs and the influence of the upper body to explain vertical jump performance. It was found, that 300 intermittent CMJs lead to explicit reduction in vertical jump performance (jump height: − 11%), and a significant decrease of knee (− 15%) and ankle joint power (− 17%) as well as a non-significant decrease of hip joint power (− 3%), as well as a significant increase of the vertical velocity of the upper body (+ 9%) allowing our hypothesis to be partially accepted.

Even earlier, the literature has shown that a decrease in jump height can be attributed to jump-induced fatigue (McNeal et al. 2010; Howell et al. 2001; Fábrica et al. 2013; Bosco et al. 1983), which decreases parameters, such as P_CoM_ (current study: −14%), GRF_Max_ (−13%), and RFD_CoM_ (−32%) (Dal pupo et al. 2012; Harman et al. 1990; Luhtanen and Komi 1978; Linthorne 2001; McLellan et al. 2011; Aragón-Vargas and Gross 1997). However, the overall decrease in jump height seems to be fatigue protocol specific, as our 11% decrease in jump height was lower than protocols with a continuous jump-induced fatigue (-30%) (Rodacki et al. 2001) or selective muscle fatigue of knee extensors (-14%) (Rodacki, André Luiz Felix et al. 2002) or plantar flexors (-18%) (Bobbert et al. 2011). A comparable fatigue protocol by Pereira et al. (2009b) with 200 intermittent CMJs with a rest interval of about 8.6 s found a 5% decrease, which is similar to the decrease (− 7%) after 200 CMJs in our study with a rest interval of 8 s. Figure 2 shows that the jump height increased during the first 50 attempts before falling. After about 100 attempts, the jump height is similar to that at the beginning. At this point, the P_CoM_ and the knee and ankle power have dropped, quite significantly in the case of the ankle, but the jump height has been maintained, suggesting that factors other than forces are contributing to performance. Future investigations should examine whether the slight increase (+ 2%) in jump height from the beginning until approximately to the 50th CMJ is a motivation-dependent or if this is due to optimized inter- and intra-coordination of the lower limb muscles.

Our results amplify the findings from the literature, that the capacities of the knee extensors and ankle plantar flexors seem to be more important than the hip extensors for vertical jump performance (Nagano and Gerritsen 2001; Cheng 2008). Following the implemented jumping-induced fatigue protocol, we found a clear redistribution in joint power from the knee (40.1–38.5%) and ankle joint (28.7–27.2%) toward the hip joint (31.2–34.3%). We determined the EMA for the joints of the lower limbs to describe the leverage characteristics of the investigated joint. Our calculated EMA values are comparable with the literature (Farris et al. 2016; Monte et al. 2021). Our jumping-based fatigue protocol led to a decrease in the EMA of hip by 5% (*p* < 0.039, *d* = 0.489) along with slight but not significant (*p* > 0.050) changes in EMA of the knee and ankle joint. This was largely because the external lever arm at the hip and hip joint angle increased significantly (*p* < 0.050), while hip joint torque did not change significantly (*p* > 0.050). As the EMA of the hip decreased significantly, it can be assumed that the effort required to perform the jump increased between CMJ_Start_ and CMJ_End_. In contrast, the EMA of the knee (+ 8%) and ankle (+ 3%) increased slightly, because the muscles were probably no longer able to provide the effort required for the jump. This can also be explained by the significantly decreased joint torque of the knee (− 11%) and ankle (− 8%) and the associated lower internal muscle force (knee: − 18%; ankle: − 16%). Although we found a comparable level of internal muscle force for knee (CMJ_Start_: 3255 N ± 1033 N; CMJ_End_: 2681 N ± 965 N) and ankle (3349 N ± 1020 N; 2818 N ± 952 N), the relative proportion of estimated active muscle volume is about 1.7 times higher at the knee than at the ankle, which could be due to the knee extensors having longer fibers than the ankle plantar flexors (Wickiewicz et al. 1983). The active muscle volume at the knee (25–24%) and ankle (15–14%) decreased slightly, while the active muscle volume at the hip increased slightly from 60 to 62% of the total volume.

This redistribution from the knee and ankle joint to the hip joint could be possibly related to individual muscle morphology, particularly differences in the distribution of muscle fiber types. The high proportion of type-II muscle fibers of the vastus lateralis (46–74% type-II muscle fibers) (Johnson et al. 1973; Inbar et al. 1981; Harridge et al. 1996; Garrett et al. 1984; Elder et al. 1982; Edgerton et al. 1975) may suggest that the knee extensors are able to produce higher and faster force, but probably have lower fatigue resistance compared to the gluteus maximus (52–68% type-I muscle fibers) (Sirca and Susec-Michieli 1980; Johnson et al. 1973), which was also observed as a decreased knee joint torque (− 11%) and power (−15%) in our study. Even though the largest ankle plantar flexor, the soleus muscle, is shown to be mostly slow-twitch type-I (63–89% type-I muscle fibers) (Johnson et al. 1973; Harridge et al. 1996; Elder et al. 1982; Edgerton et al. 1975; Dahmane et al. 2005), the fast-twitch type-II gastrocnemii (40–62% type-II muscle fibers) (Dahmane et al. 2005; Edgerton et al. 1975; Johnson et al. 1973), may be still more likely to fatigue, as we found a decrease in ankle joint torque (− 8%) and power (− 17%). Nevertheless, a discussion of the influence by the individual muscle morphology remains highly speculative without a detailed analysis of muscle morphology (e.g., muscle biopsy), wherefore future investigations should examine the relationship between muscle morphology and the mechanical power redistribution at the lower limb joints due to jump-induced fatigue protocols.

The fatigue protocol used in this study led to a significant decrease of the knee and ankle joint torques and power, whereby the knee joint torques were possibly more affected by the jump-induced fatigue through a higher accumulation of metabolites due to a relatively higher active muscle mass in comparison to the ankle plantar flexors. Generally, fatigue is identified as a reduction in the ability of muscle to produce force or power (Bigland-Ritchie and Woods 1984; Søgaard et al. 2006; Bigland-Ritchie et al. 1983; Barry and Enoka 2007; Gandevia 2001), which is induced by an increased physical load on the cardiovascular system and through an accumulation of metabolites (Fitts 1994; Gaesser and Poole 1996). The peripheral (muscular) fatigue is characterized by exercise-related change in the blood, extracellular fluid, and within muscle fibers. Within the muscle fibers, inorganic phosphate, H^+^ ions and Mg^2+^ ions, among others, accumulate in the sarcoplasm, and the Ca^2+^ release from the sarcoplasmic reticulum is inhibited by the accumulation of inorganic phosphate (Ament and Verkerke 2009). These changes are typical of fatiguing stress. Therefore, we assume that these changes also occurred within the muscle fibers of our participants. On the other hand: exercise-related changes in the blood, more specifically an accumulation of lactate and hydrogen ions (protons), are further changes that occur as a result of peripheral fatigue. The accumulation of hydrogen ions is partly buffered, such that there is an increased liberation of carbon dioxide from bicarbonate. As a result, the respiratory quotient increases and the cardiovascular system powers up to ensure sufficient blood flow to the body (Ament and Verkerke 2009). In the current study, we found after 100 CMJs a heart rate of 168 ± 11 BPM and lactate concentration of 3.05 ± 1.38 mmol·l^−1^, which did not significantly further change after continuing the CMJs. The determined blood lactate concentration in the present study was near to the aerobic-anaerobic 4 mmol·l^−1^ threshold, often defined as the transition point from aerobic to partly anaerobic energy metabolism (Mader et al. 1976). Hence, the energy supply using the oxidative pathways seems to undertake a dominate role during intermittent 300 CMJs and the role of the glycolytic pathway declines by glycolytic deficiency. These results indicate that the participants were physiologically and metabolically stressed during this endurance exercise. Accordingly, we speculate that 300 intermittent CMJs with an 8 s rest interval resulted in the accumulation of metabolites in the leg-extensor muscles dependent of the individual muscle fiber distribution, thereby changing the mechanical power distribution at the lower limbs and affecting vertical jump performance. Further investigations are required to clarify this presumption. In addition, central (neural) fatigue during jumping tasks should be investigated to determine whether the motor neuronal drive is influenced by reflex effects of muscle afferents (Ament and Verkerke 2009).

When investigating more closely the effect of the motion of the upper body, we found a significant increase in the hip flexion angle (+ 5%), a larger lever arm at the hip joint (+ 6%) as well as a higher angular momentum during the breaking (+ 11%) and propulsion phase (+ 18%) (Table 1) and an increase in the maximal vertical velocity of upper body segment (+ 9%). We found an 8% reduction in internal muscle force of the hip extensors. We assume that the upper body generated an additional torque at the hip joint, as it has tilted further forward during the braking phase (+ 5° hip flexion angle), and thus, more angular momentum could be generated in the propulsion phase by longer acceleration path to counteract the possible fatigue at the hip joint. This suggests that the participants performed additional mechanical work on the CoM using their erector spinae during CMJs to compensate the decrease in the joint power of the lower limb extensors (Blache and Monteil 2014, 2015; Bobbert and van Soest 2001). The absence of the typical symptoms of fatigue in the upper body could be related to the relatively fatigue resistant erector spinae (from muscle fiber characteristics predominantly slow-twitch type-I fibers) and its function as a postural muscle (Agten et al. 2020; Mannion et al. 1997). Accordingly, further studies could examine the differences in maximal shortening velocities of erector spinae and lower limb extensors in a non-fatigued and fatigued state, as well as consider individual muscle fiber characteristics.

## Limitations

First, we did not determine the individual best performance in vertical jumping before the fatigue protocol. Second, the participants could have been differently motivated to perform each of all 300 CMJs, which required voluntary executed maximal effort muscle contractions under fatigue effects. Third, we did not determine the isometric or isokinetic force capacities nor the muscle fiber distribution of the lower limb extensors. Fourth, the use of electromyography might have provided complementary information about the muscle activation changes during the fatigue protocol. Fifth, the use of magnetic resonance imaging could have been determined the individual internal lever arm for each participant to determine the EMA more individually.

## Conclusion

Our findings demonstrate that a jump-induced fatigue protocol of 300 intermittent CMJs with an 8 s rest interval led to a reduction in vertical jump performance and resulted in a decreased knee and ankle joint power. Although a decrease in internal muscle force and an increase in relative active muscle volume of the hip extensors were observed, no changes in the hip joint torque or power were detected. This is possibly due to additional torque created by the trunk extensor muscles, which increased the angular momentum and maximal vertical velocity of the upper body segment.

We suggest that a targeted exercise to enhance trunk and leg-extensor muscles, especially knee extensor and ankle plantar flexor muscles, would improve vertical jump and athletics performance in sports requiring constant ability to jump. The mechanical influence of the trunk extensor muscles should not be neglected in vertical jump performance, especially under fatigue conditions.
